# Supplementary material for: Rice GA3ox1 modulates pollen starch granule accumulation and pollen wall development
Source: PLoS One. 2023 Oct 9;18(10):e0292400. doi: 10.1371/journal.pone.0292400 (PMC10561864; doi:10.1371/journal.pone.0292400)
Supplement: S1 Fig — A) The gene structure of OsGA3ox1 showing exons (boxes), introns (lines), untranslated regions (UTRs) and sequences of sgRNA (red letters are the PAMs) at their target site (red vertical line). The primers (3ox1-CF and 3ox1-CR) used for DNA amplification and the expected sizes (in bp) of the PCR products are shown. A total of 1964 bp is used to draw this diagram. B) The template vector pRGEB32 for CRISPR/Cas9 constructs. Cas9 driven by the ubiquitin promoter (Pubi), gRNA (red) and scaffold (blue) driven by the U3 promoter (POsU3) and hygromycin phosphotransferase (hpt) driven by the caMV35S promoter are indicated. The primers (arrows) used for detecting the T-DNA construct (F and R) and hpt gene (hpt-F and hpt-R) are shown. C) Characterization of 8 Cas9-induced transgenic T0 lines. PCR products were amplified from their construct and target gene regions using the primers indicated in B) and A), respectively. After PCR product sequencing, each of their sequencing results, either insertion (In) “+” or deletion (Del) “-“, is indicated below each line using Arabic numerals separated by a slash (±n/±n) to represent their various diploid allelic genotypes. The superscript star sign (*) indicates that additional unidentified sequence modifications may exist in the line. The T1 seed-setting rate (SSR) for each line was measured and is provided below. D) The detailed In/Del sequences around the target site for each Cas9-induced line. Sequence data were obtained by Sanger sequencing using either the PCR products directly and/or plasmid DNA with cloned PCR products through the TA vector. E) Derived amino acid sequences based on the DNA sequences identified for various allelic templates. Deletions of amino acids are indicated with dashed lines. The “-3” deletions were all the same among different lines. The conserved amino acid sequences for all alleles at the N-terminal are indicated with red print, the replaced amino acid sequences due to frameshift are indicated with blue pr [file pone.0292400.s001.pptx]

## Slide 1
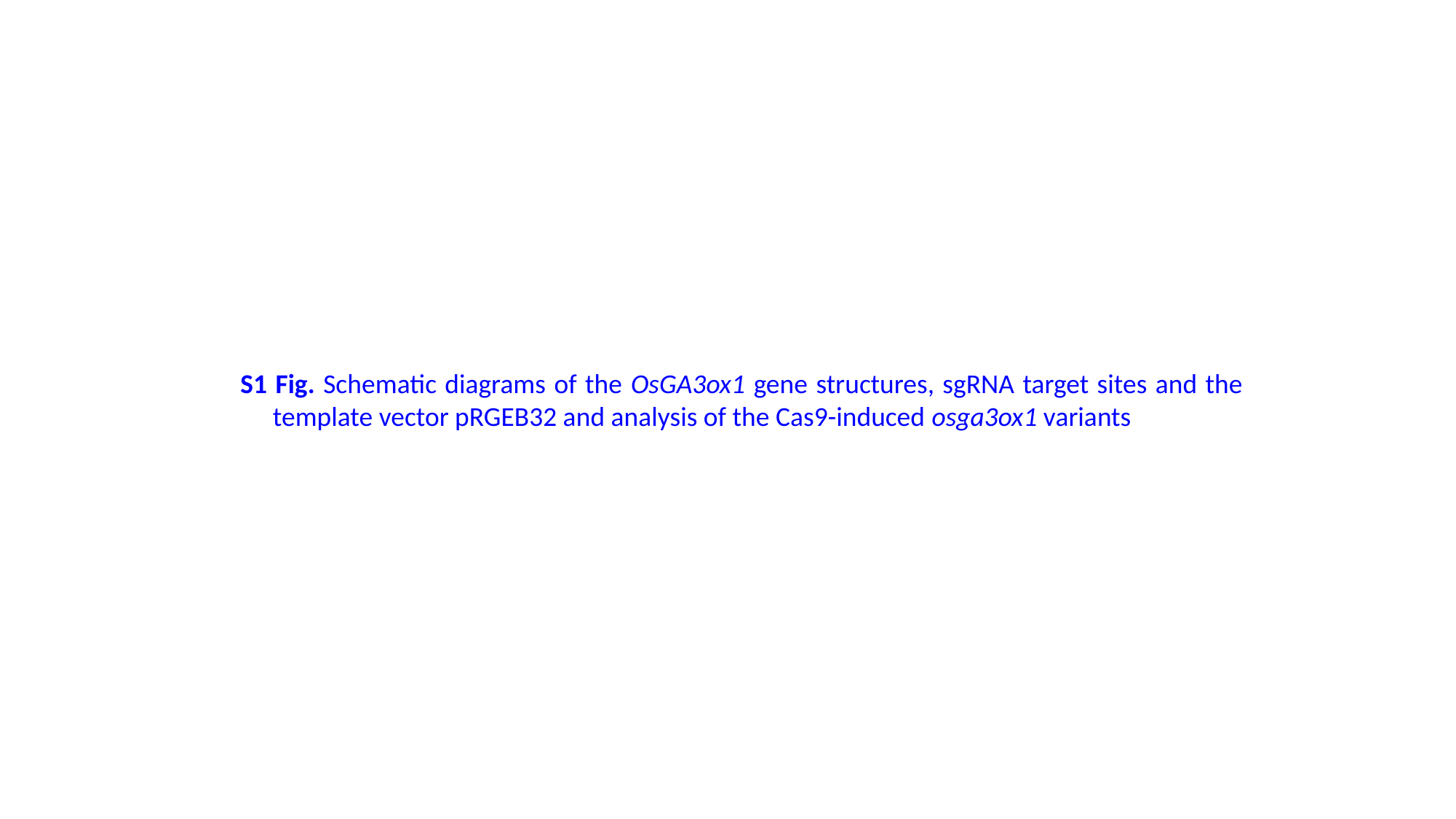

S1 Fig. Schematic diagrams of the OsGA3ox1 gene structures, sgRNA target sites and the template vector pRGEB32 and analysis of the Cas9-induced osga3ox1 variants

## Slide 2
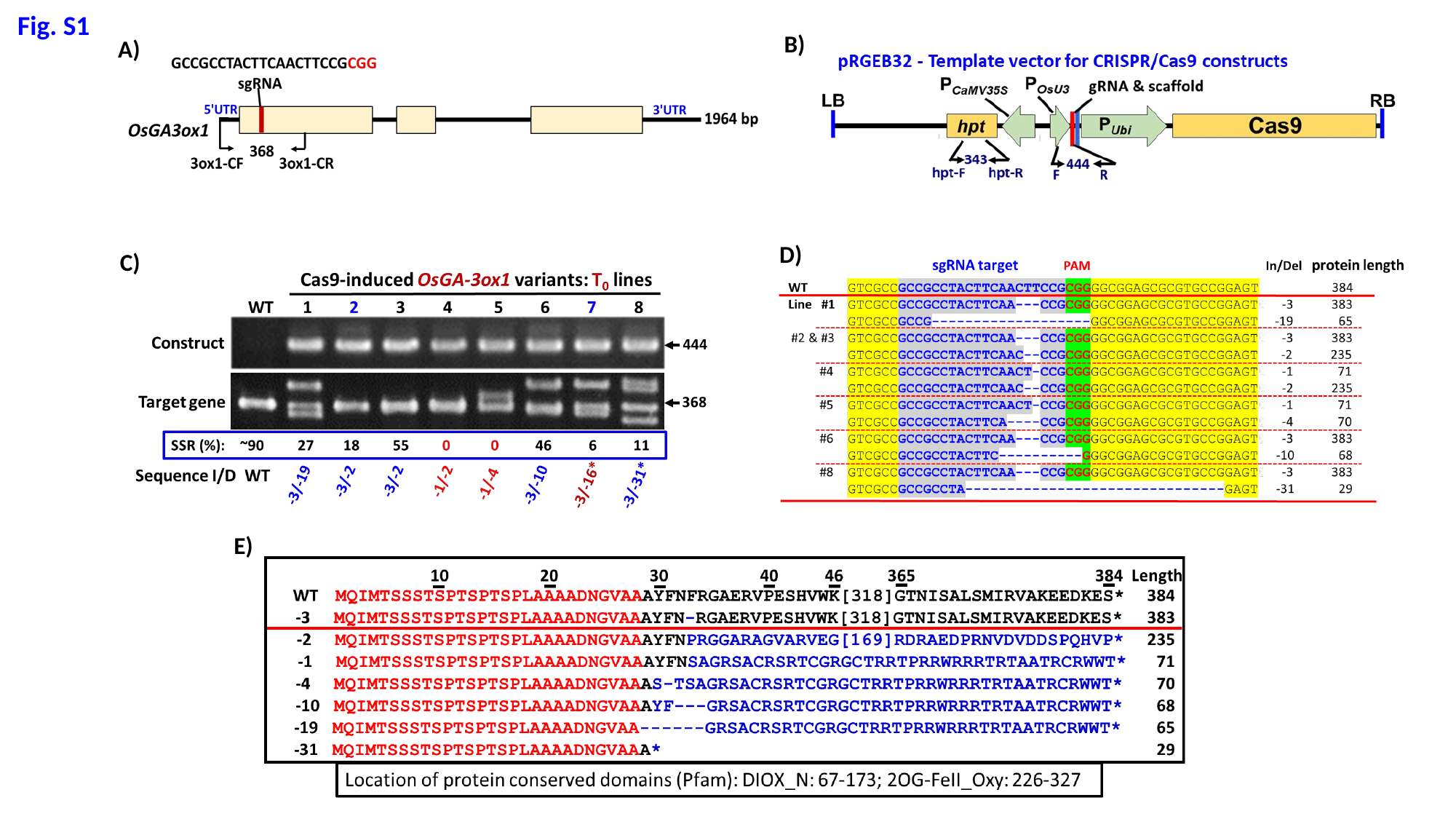

Fig. S1
B)
A)
D)
C)
E)
