# Supplementary figures and images for: Rice GA3ox1 modulates pollen starch granule accumulation and pollen wall development
Source: PLoS One. 2023 Oct 9;18(10):e0292400. doi: 10.1371/journal.pone.0292400 (PMC10561864; doi:10.1371/journal.pone.0292400)

## Slide 1
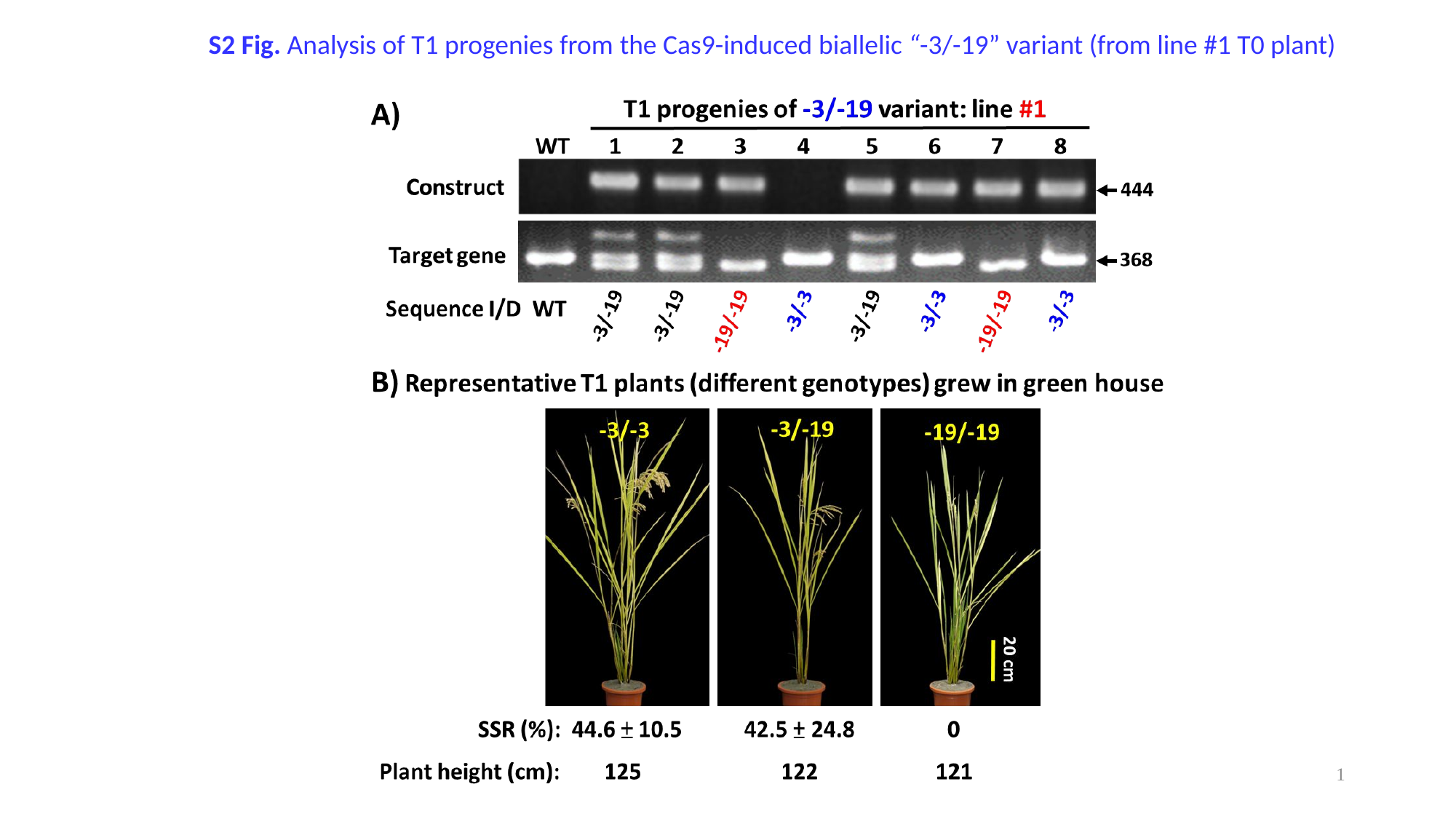

S2 Fig. Analysis of T1 progenies from the Cas9-induced biallelic “-3/-19” variant (from line #1 T0 plant)
1

Supplement: S2 Fig — A) PCR product analysis of 8 transgenic T1 progenies for their constructs and target genes. Plants were segregated into “-3/-3”, “-19/-19” and “-3/-19” recognized by gel patterns and confirmed by sequencing. The sequence In/Del for each variant is indicated. B) Representative T1 plants from different genotypes with their plant height and seed-setting rate (SSR). Plants were grown in the greenhouse. (PPTX) [file pone.0292400.s002.pptx]

## Slide 1
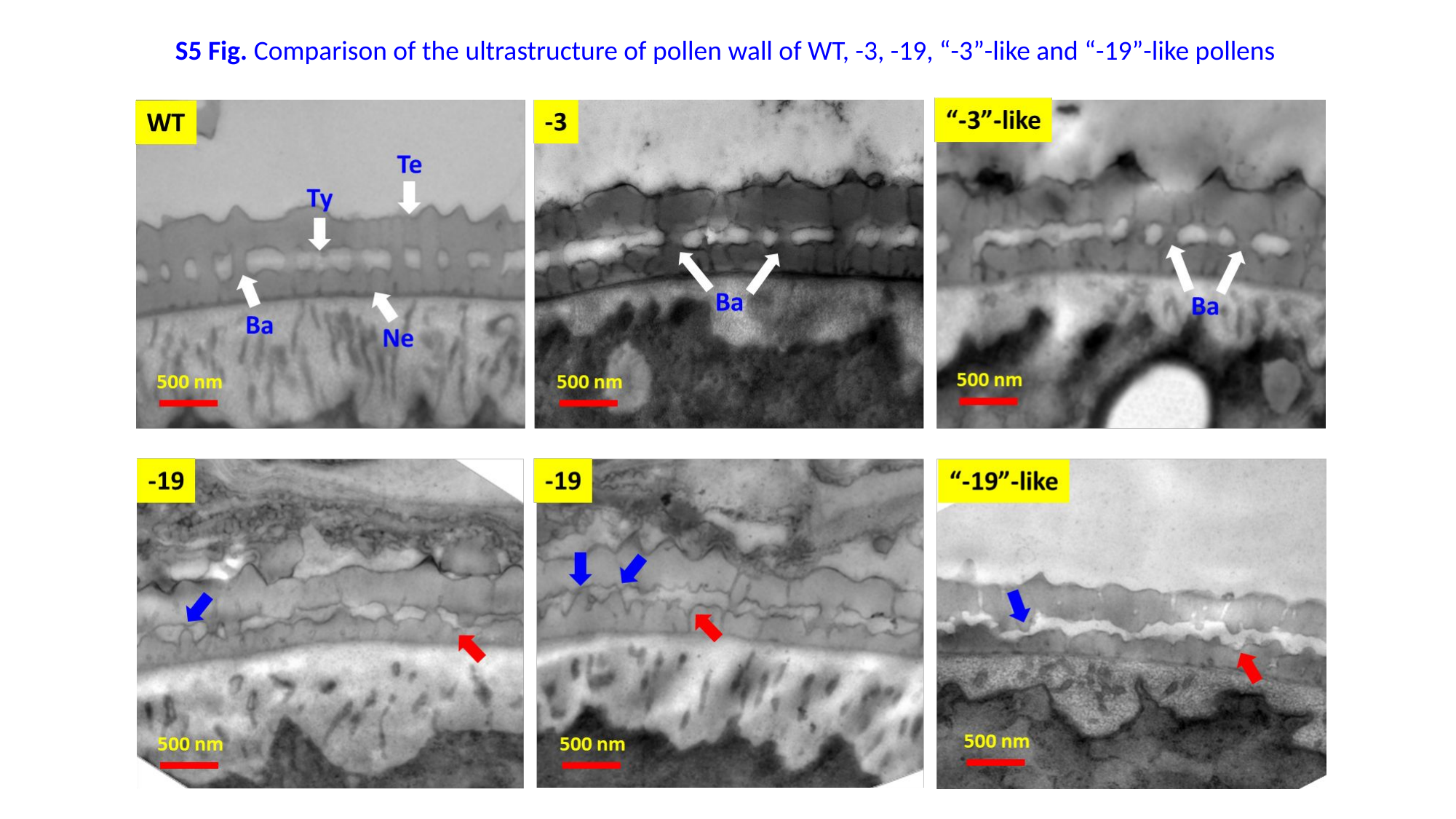

S5 Fig. Comparison of the ultrastructure of pollen wall of WT, -3, -19, “-3”-like and “-19”-like pollens

Supplement: S5 Fig — The “-3” and “-19” pollens were from homozygous “-3/-3” and “-19/-19” anther, respectively. The “-3”-like and “-19”-like pollens were from the heterozygous “-3/-19” mutant. The structure of exine, including tectum (Te), tryphine (Ty), baculum (Ba) and nexine (Ne), are indicated in the WT pollen. The abnormal formation of baculum (blue arrow) and tryphine (red arrow) in “-19” and “-19”-like pollen are pointed. Bar = 500 nm. (PPTX) [file pone.0292400.s005.pptx]
