# Supplementary material for: Rice GA3ox1 modulates pollen starch granule accumulation and pollen wall development
Source: PLoS One. 2023 Oct 9;18(10):e0292400. doi: 10.1371/journal.pone.0292400 (PMC10561864; doi:10.1371/journal.pone.0292400)
Supplement: S3 Fig — A) PCR product analysis of 24 T2 progenies from the “3/-19” biallelic variant (T1 plant #2 in S2 Fig). T2 Plants were segregated to 11:11:2 = “-3/-3”:“-3/-19”:“-19/-19” based on the gel patterns and confirmed by sequencing. The sequence In/Del for each plant is indicated below. B) Representative T2 plants from different genotypes “-3/-3”: “-3/-19”: “-19/-19”. Plants were grown in the paddy field. Bar = 20 cm. (PPTX) [file pone.0292400.s003.pptx]

## Slide 1
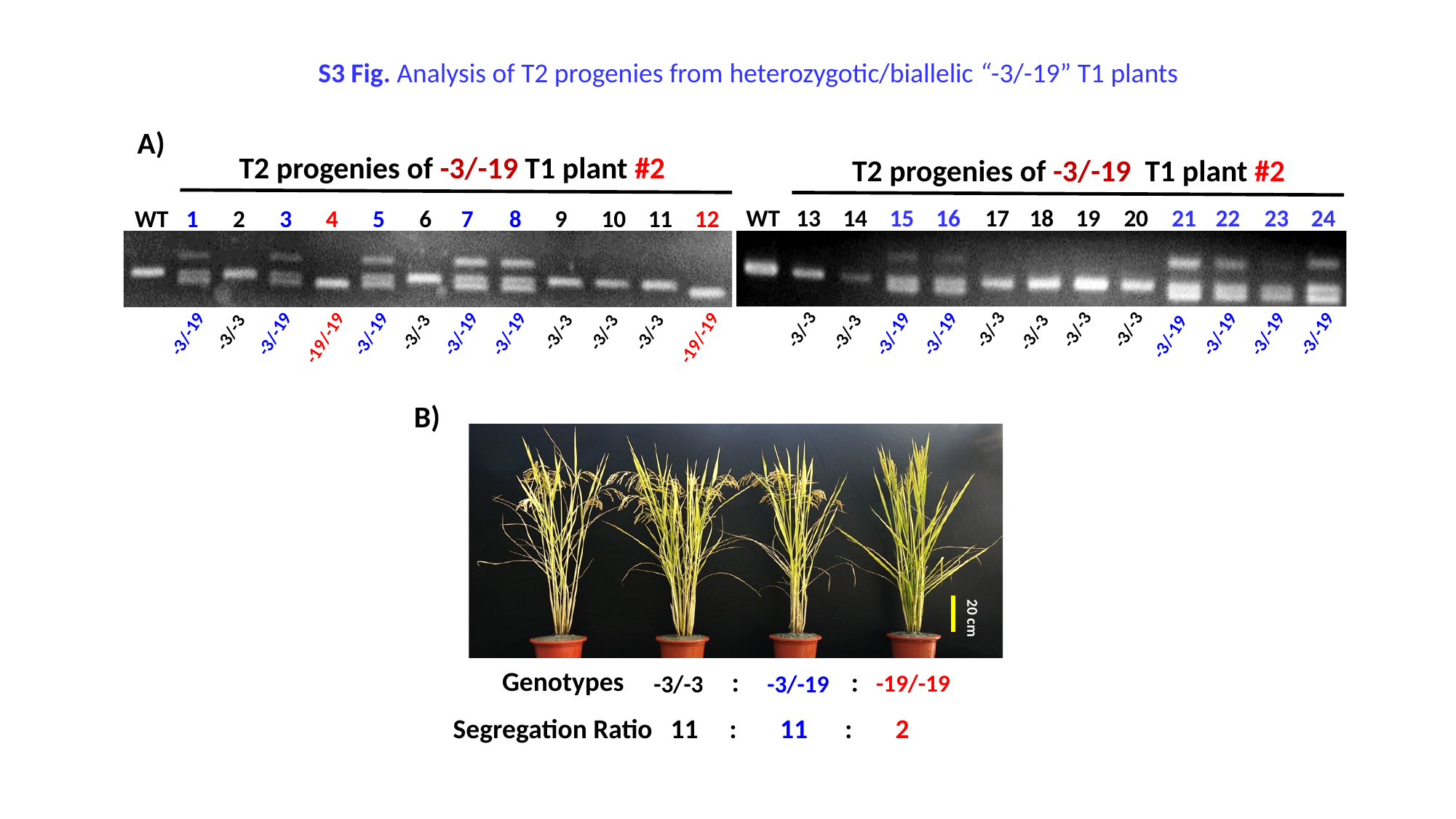

S3 Fig. Analysis of T2 progenies from heterozygotic/biallelic “-3/-19” T1 plants
A)
T2 progenies of -3/-19 T1 plant #2
WT
1
2
3
4
5
6
7
8
9
10
11
12
-3/-3
-3/-3
-3/-3
-3/-3
-3/-3
-3/-19
-3/-19
-3/-19
-3/-19
-3/-19
-19/-19
-19/-19
T2 progenies of -3/-19 T1 plant #2
WT
13
14
15
16
17
18
19
20
21
22
23
24
-3/-3
-3/-3
-3/-3
-3/-3
-3/-3
-3/-3
-3/-19
-3/-19
-3/-19
-3/-19
-3/-19
-3/-19
B)
Genotypes
:
:
-19/-19
-3/-3
-3/-19
Segregation Ratio
11 : 11 : 2
20 cm
