# Supplementary material for: Rice GA3ox1 modulates pollen starch granule accumulation and pollen wall development
Source: PLoS One. 2023 Oct 9;18(10):e0292400. doi: 10.1371/journal.pone.0292400 (PMC10561864; doi:10.1371/journal.pone.0292400)
Supplement: S4 Fig — The anther microscopic sections of WT, heterozygous (“-3/-19” and “-3/-10”) and homozygous (“-3/-3”, “-19/-19” and “-2/-2”) osga3ox1 variants are shown. Two types of pollen grains, the normal pollen grain (“-3”-like, blue arrows) and abnormal/starch-less pollen grain (“-10”-like and “-19”-like, red arrows) are observed within pollen sacs of “-3/-19” and “-3/-10” heterozygous mutants. The “-3”-like and “-19”-like pollens can be visually identified, examples of different pollen type from each sac are indicated with corresponding base deletion symbols. A) one anther sac with better resolution, Bar = 20 μm. B) sections with 4 anther sacs, Bar = 70 μm. (PPTX) [file pone.0292400.s004.pptx]

## Slide 1
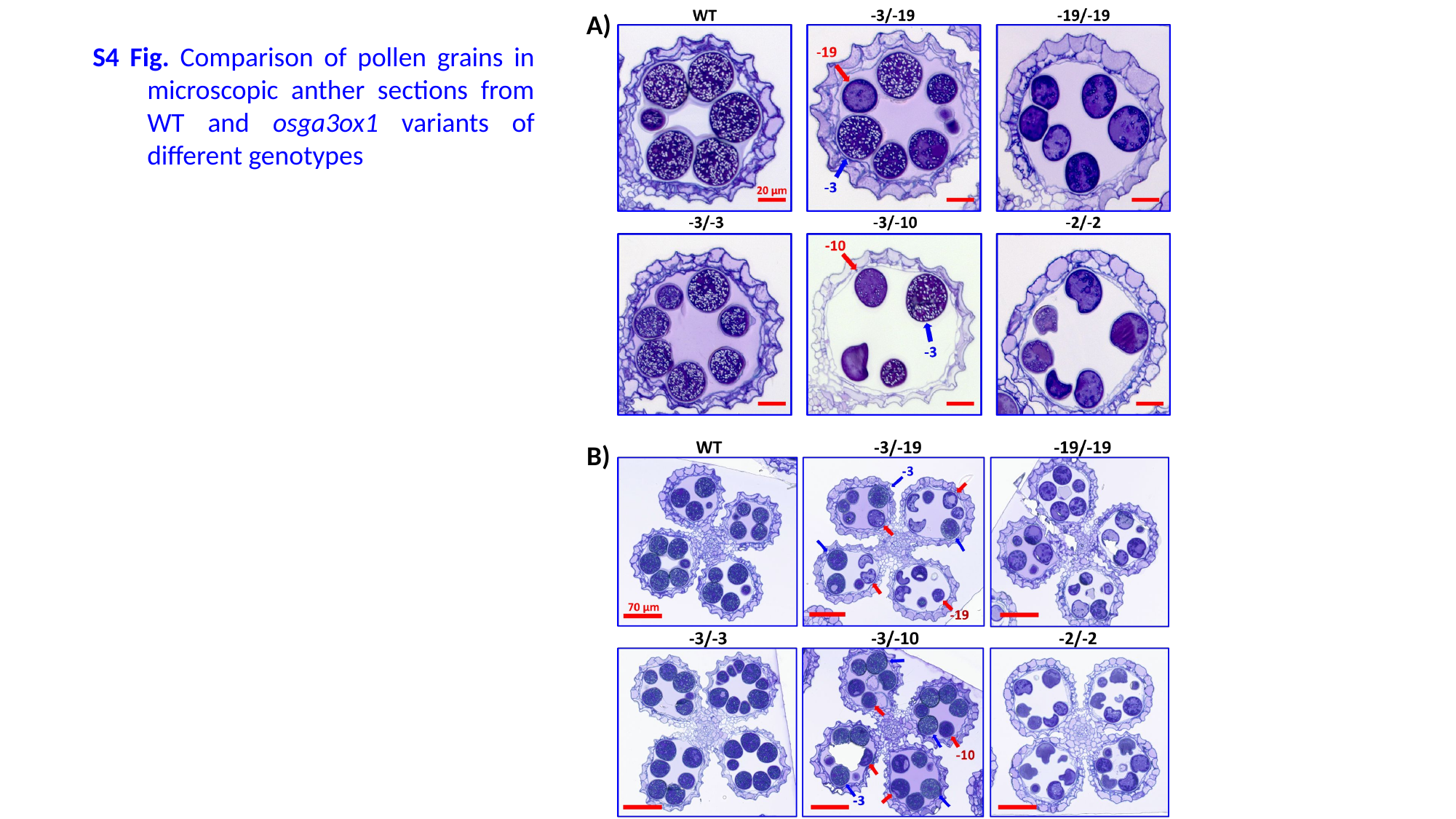

A)
S4 Fig. Comparison of pollen grains in microscopic anther sections from WT and osga3ox1 variants of different genotypes
B)
