# Supplementary material for: Rice GA3ox1 modulates pollen starch granule accumulation and pollen wall development
Source: PLoS One. 2023 Oct 9;18(10):e0292400. doi: 10.1371/journal.pone.0292400 (PMC10561864; doi:10.1371/journal.pone.0292400)
Supplement: S6 Fig — Expression analysis of the OsGAMYB, OsTDR, and OsbHLH142 pollen development genes in WT and the “-10/-10” and “-19/-19” ga3ox1 knockout mutants. The significant differences between WT and knockout mutants were determined by Student’s t test. *p<0.05, **p<0.01, ***p<0.001. (PPTX) [file pone.0292400.s006.pptx]

## Slide 1
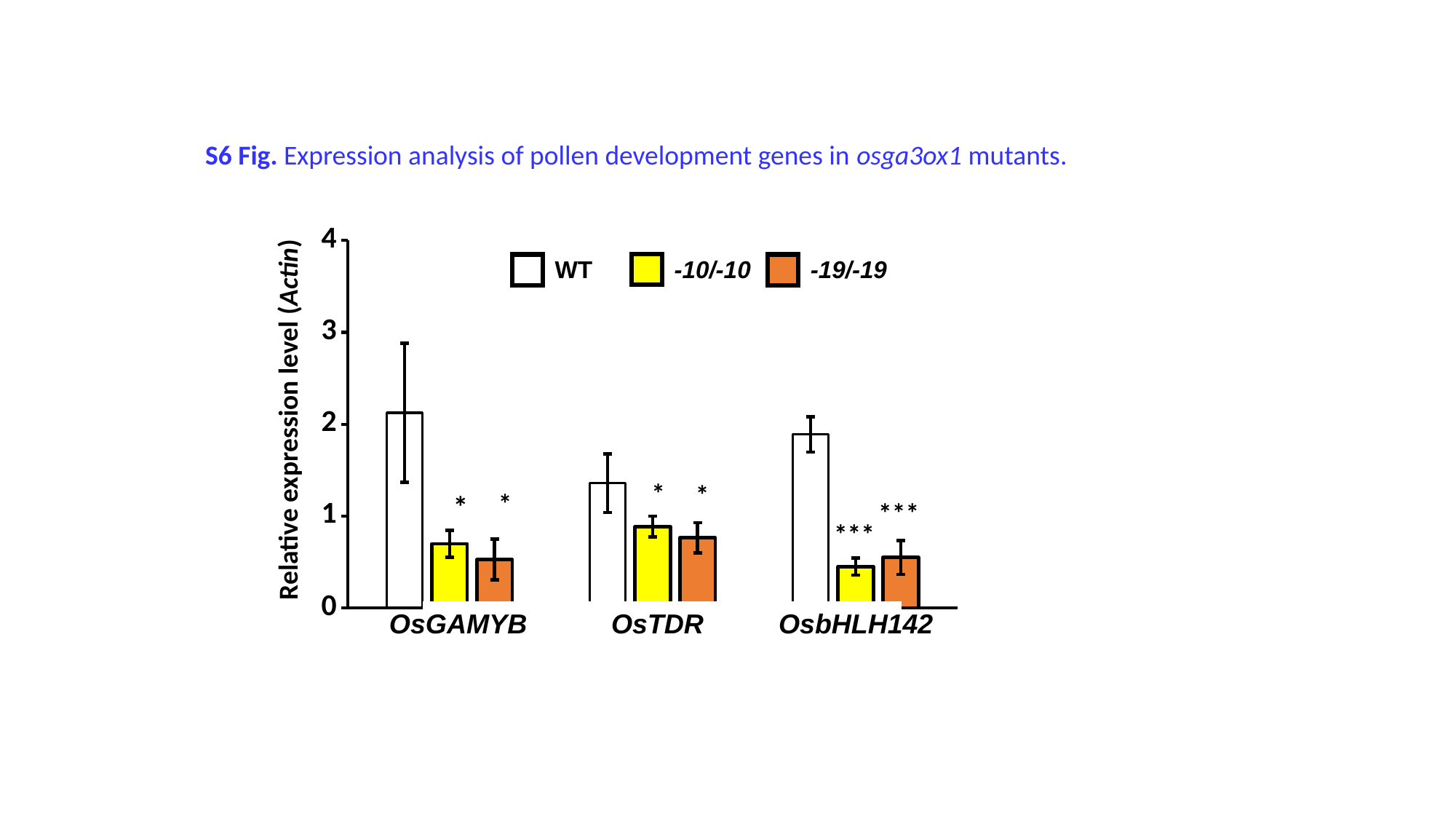

S6 Fig. Expression analysis of pollen development genes in osga3ox1 mutants.
### Chart
| Category | | | |
|---|---|---|---|OsbHLH142
OsGAMYB
OsTDR
Relative expression level (Actin)
*
*
*
*
***
***
WT
-10/-10
-19/-19
