# Supplementary material for: Rice GA3ox1 modulates pollen starch granule accumulation and pollen wall development
Source: PLoS One. 2023 Oct 9;18(10):e0292400. doi: 10.1371/journal.pone.0292400 (PMC10561864; doi:10.1371/journal.pone.0292400)
Supplement: S1 Table — (PPTX) [file pone.0292400.s007.pptx]

## Slide 1
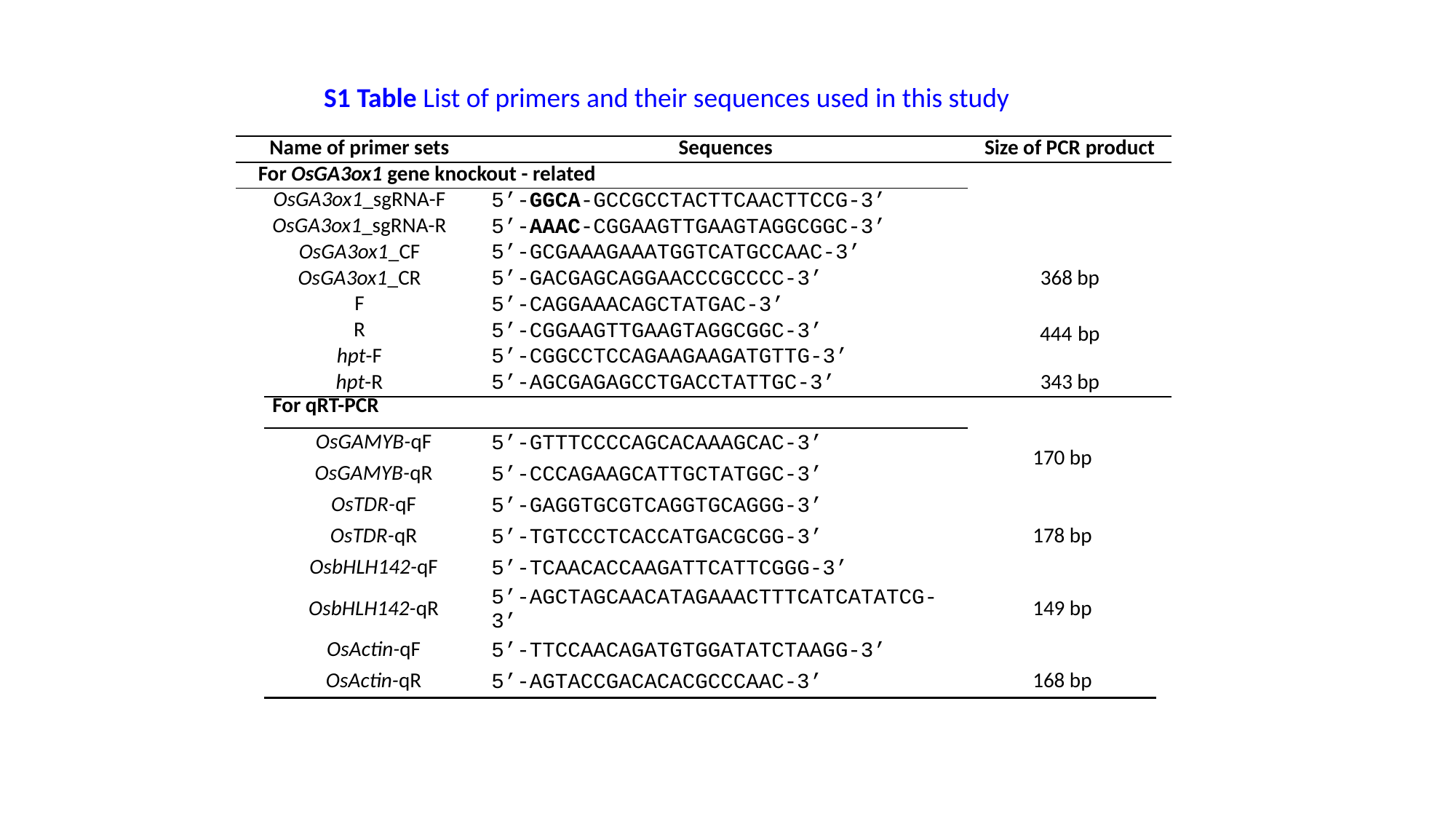

S1 Table List of primers and their sequences used in this study
| Name of primer sets | | Sequences | Size of PCR product | |
| --- | --- | --- | --- | --- |
| For OsGA3ox1 gene knockout - related | | | | |
| OsGA3ox1\_sgRNA-F | | 5’-GGCA-GCCGCCTACTTCAACTTCCG-3’ | | |
| OsGA3ox1\_sgRNA-R | | 5’-AAAC-CGGAAGTTGAAGTAGGCGGC-3’ | | |
| OsGA3ox1\_CF | | 5’-GCGAAAGAAATGGTCATGCCAAC-3’ | | |
| OsGA3ox1\_CR | | 5’-GACGAGCAGGAACCCGCCCC-3’ | 368 bp | |
| F | | 5’-CAGGAAACAGCTATGAC-3’ | | |
| R | | 5’-CGGAAGTTGAAGTAGGCGGC-3’ | 444 bp | |
| hpt-F | | 5’-CGGCCTCCAGAAGAAGATGTTG-3’ | | |
| hpt-R | | 5’-AGCGAGAGCCTGACCTATTGC-3’ | 343 bp | |
| | For qRT-PCR | | | |
| | OsGAMYB-qF | 5’-GTTTCCCCAGCACAAAGCAC-3’ | 170 bp | |
| | OsGAMYB-qR | 5’-CCCAGAAGCATTGCTATGGC-3’ | | |
| | OsTDR-qF | 5’-GAGGTGCGTCAGGTGCAGGG-3’ | | |
| | OsTDR-qR | 5’-TGTCCCTCACCATGACGCGG-3’ | 178 bp | |
| | OsbHLH142-qF | 5’-TCAACACCAAGATTCATTCGGG-3’ | | |
| | OsbHLH142-qR | 5’-AGCTAGCAACATAGAAACTTTCATCATATCG-3’ | 149 bp | |
| | OsActin-qF | 5’-TTCCAACAGATGTGGATATCTAAGG-3’ | | |
| | OsActin-qR | 5’-AGTACCGACACACGCCCAAC-3’ | 168 bp | |
