# Supplementary material for: Rice GA3ox1 modulates pollen starch granule accumulation and pollen wall development
Source: PLoS One. 2023 Oct 9;18(10):e0292400. doi: 10.1371/journal.pone.0292400 (PMC10561864; doi:10.1371/journal.pone.0292400)
Supplement: S1 Raw images — (PPTX) [file pone.0292400.s008.pptx]

## Slide 1
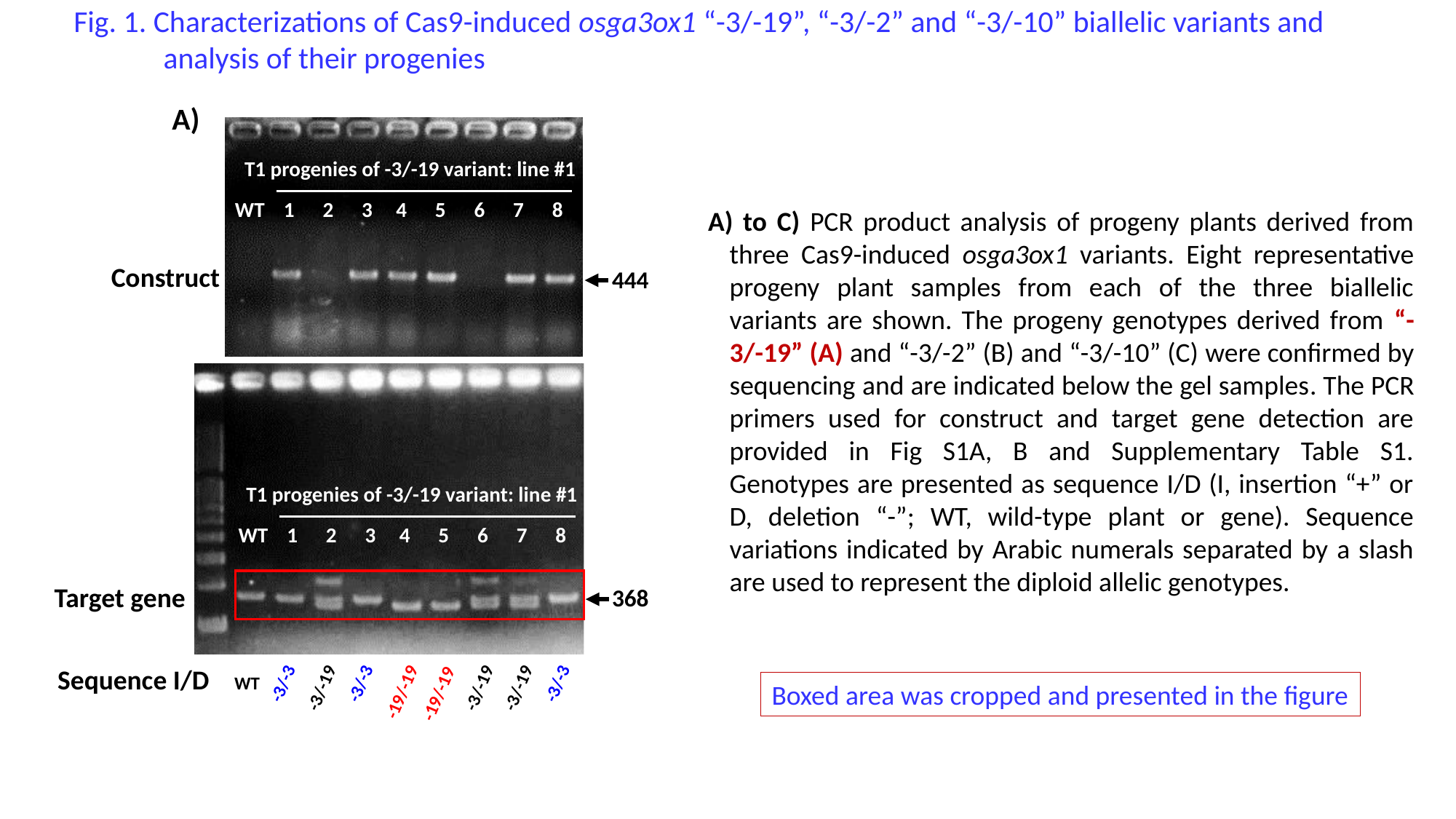

Fig. 1. Characterizations of Cas9-induced osga3ox1 “-3/-19”, “-3/-2” and “-3/-10” biallelic variants and
 analysis of their progenies
A)
T1 progenies of -3/-19 variant: line #1
WT 1 2 3 4 5 6 7 8
A) to C) PCR product analysis of progeny plants derived from three Cas9-induced osga3ox1 variants. Eight representative progeny plant samples from each of the three biallelic variants are shown. The progeny genotypes derived from “-3/-19” (A) and “-3/-2” (B) and “-3/-10” (C) were confirmed by sequencing and are indicated below the gel samples. The PCR primers used for construct and target gene detection are provided in Fig S1A, B and Supplementary Table S1. Genotypes are presented as sequence I/D (I, insertion “+” or D, deletion “-”; WT, wild-type plant or gene). Sequence variations indicated by Arabic numerals separated by a slash are used to represent the diploid allelic genotypes.
Construct
444
T1 progenies of -3/-19 variant: line #1
WT 1 2 3 4 5 6 7 8
Target gene
368
Sequence I/D WT
-3/-3
-3/-3
-3/-3
-3/-19
-3/-19
-3/-19
Boxed area was cropped and presented in the figure
-19/-19
-19/-19

## Slide 2
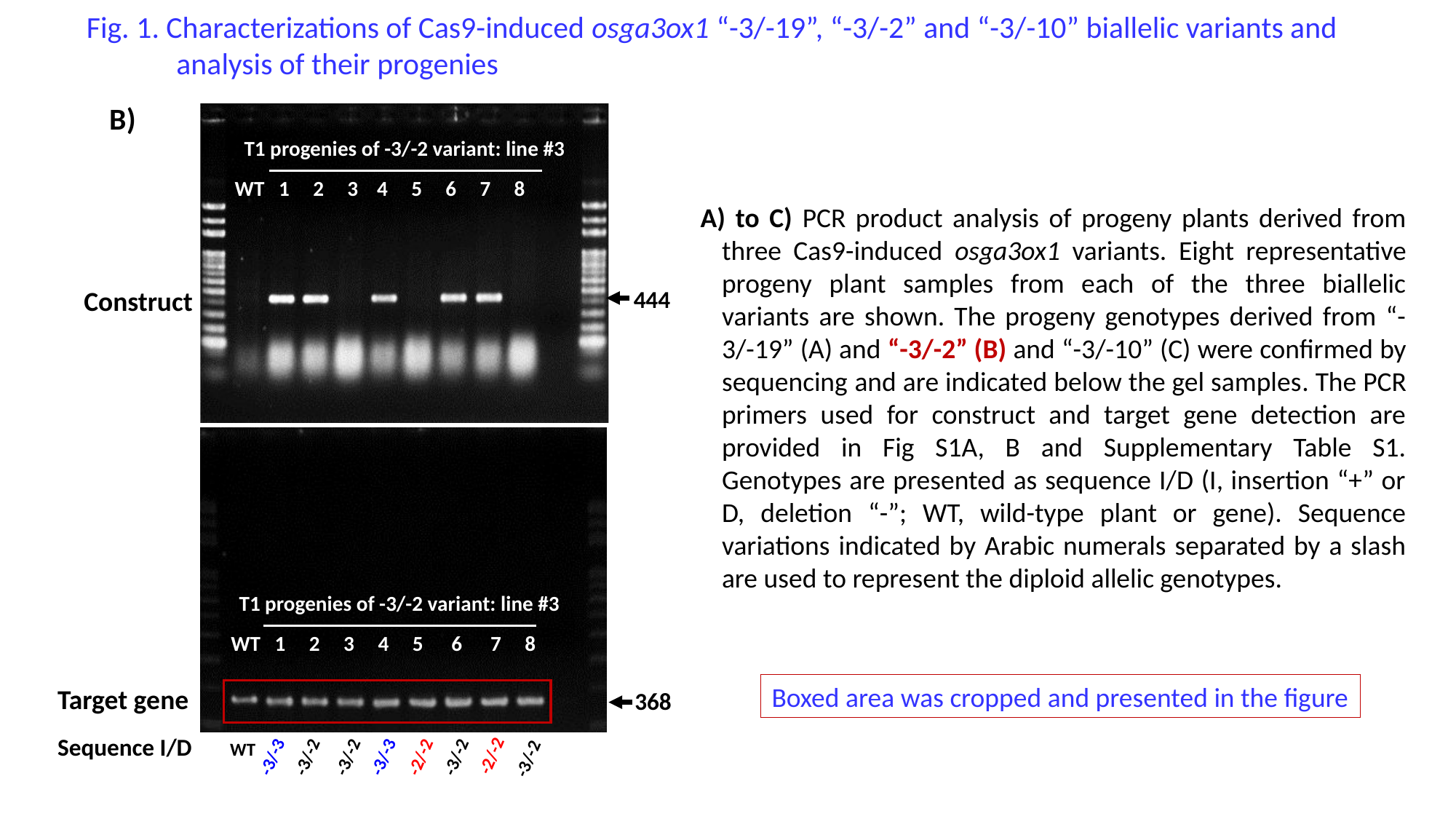

Fig. 1. Characterizations of Cas9-induced osga3ox1 “-3/-19”, “-3/-2” and “-3/-10” biallelic variants and
 analysis of their progenies
B)
Construct
444
Target gene
368
T1 progenies of -3/-2 variant: line #3
WT 1 2 3 4 5 6 7 8
Sequence I/D WT
-3/-3
-3/-2
-3/-2
-3/-3
-2/-2
-3/-2
-2/-2
-3/-2
A) to C) PCR product analysis of progeny plants derived from three Cas9-induced osga3ox1 variants. Eight representative progeny plant samples from each of the three biallelic variants are shown. The progeny genotypes derived from “-3/-19” (A) and “-3/-2” (B) and “-3/-10” (C) were confirmed by sequencing and are indicated below the gel samples. The PCR primers used for construct and target gene detection are provided in Fig S1A, B and Supplementary Table S1. Genotypes are presented as sequence I/D (I, insertion “+” or D, deletion “-”; WT, wild-type plant or gene). Sequence variations indicated by Arabic numerals separated by a slash are used to represent the diploid allelic genotypes.
T1 progenies of -3/-2 variant: line #3
WT 1 2 3 4 5 6 7 8
Boxed area was cropped and presented in the figure

## Slide 3
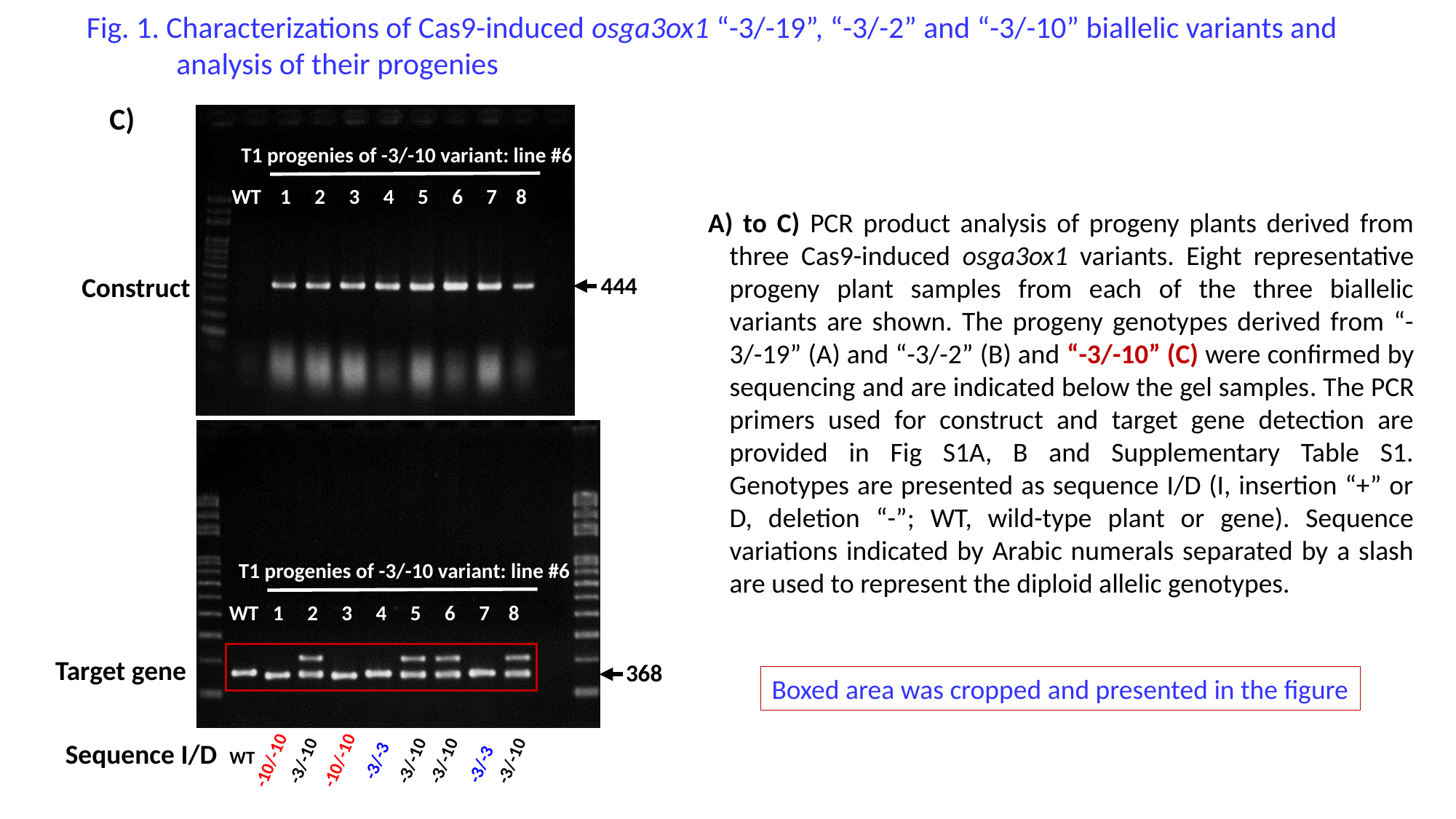

Fig. 1. Characterizations of Cas9-induced osga3ox1 “-3/-19”, “-3/-2” and “-3/-10” biallelic variants and
 analysis of their progenies
C)
T1 progenies of -3/-10 variant: line #6
WT 1 2 3 4 5 6 7 8
Construct
444
T1 progenies of -3/-10 variant: line #6
WT 1 2 3 4 5 6 7 8
Target gene
368
Sequence I/D WT
-10/-10
-3/-10
-10/-10
-3/-3
-3/-10
-3/-10
-3/-10
-3/-3
A) to C) PCR product analysis of progeny plants derived from three Cas9-induced osga3ox1 variants. Eight representative progeny plant samples from each of the three biallelic variants are shown. The progeny genotypes derived from “-3/-19” (A) and “-3/-2” (B) and “-3/-10” (C) were confirmed by sequencing and are indicated below the gel samples. The PCR primers used for construct and target gene detection are provided in Fig S1A, B and Supplementary Table S1. Genotypes are presented as sequence I/D (I, insertion “+” or D, deletion “-”; WT, wild-type plant or gene). Sequence variations indicated by Arabic numerals separated by a slash are used to represent the diploid allelic genotypes.
Boxed area was cropped and presented in the figure

## Slide 4
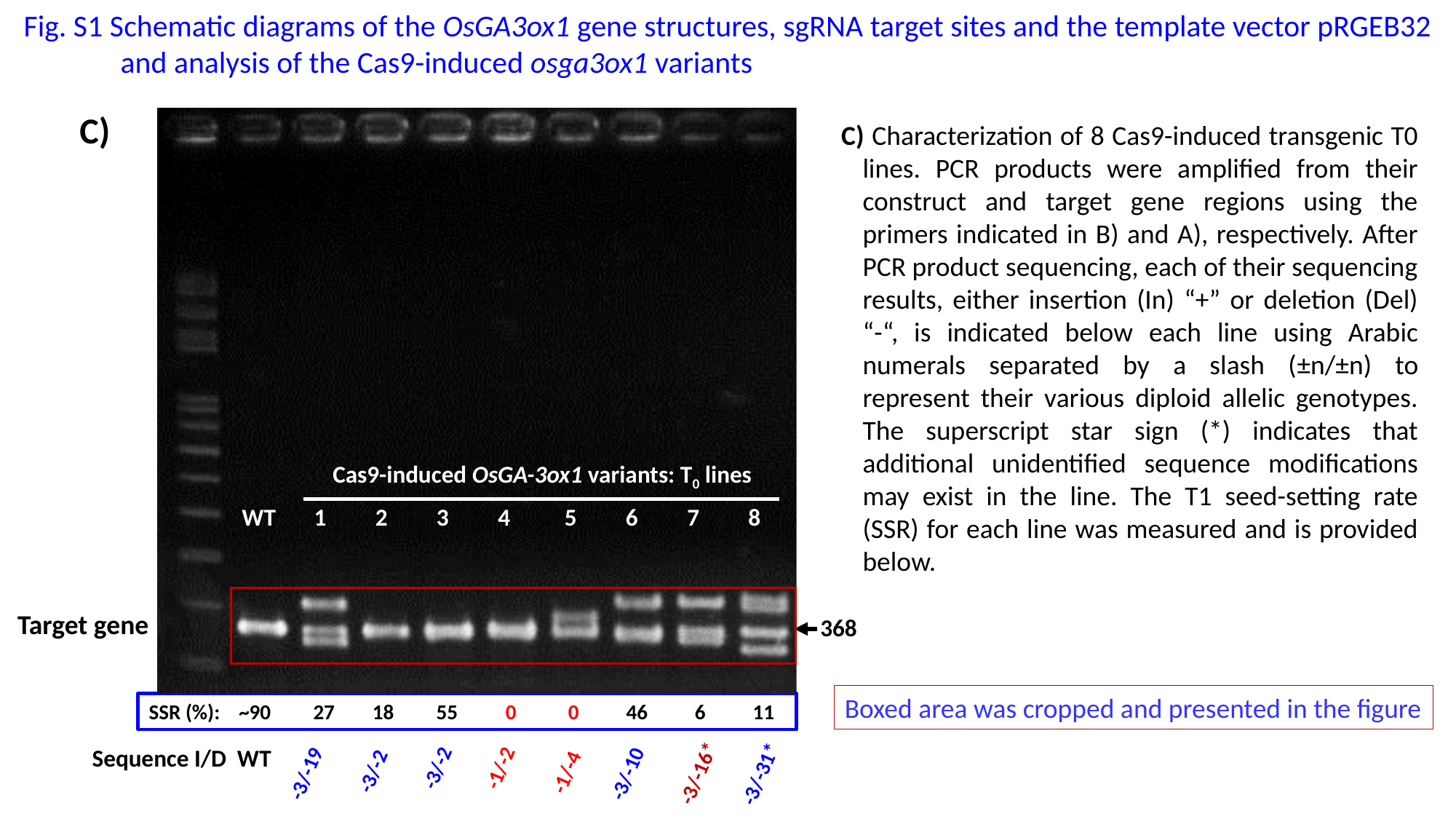

Fig. S1 Schematic diagrams of the OsGA3ox1 gene structures, sgRNA target sites and the template vector pRGEB32
 and analysis of the Cas9-induced osga3ox1 variants
C)
Cas9-induced OsGA-3ox1 variants: T0 lines
WT 1 2 3 4 5 6 7 8
Target gene
368
SSR (%): ~90 27 18 55 0 0 46 6 11
Sequence I/D WT
-3/-2
-3/-2
-1/-2
-1/-4
-3/-16*
-3/-19
-3/-10
-3/-31*
C) Characterization of 8 Cas9-induced transgenic T0 lines. PCR products were amplified from their construct and target gene regions using the primers indicated in B) and A), respectively. After PCR product sequencing, each of their sequencing results, either insertion (In) “+” or deletion (Del) “-“, is indicated below each line using Arabic numerals separated by a slash (±n/±n) to represent their various diploid allelic genotypes. The superscript star sign (*) indicates that additional unidentified sequence modifications may exist in the line. The T1 seed-setting rate (SSR) for each line was measured and is provided below.
Boxed area was cropped and presented in the figure

## Slide 5
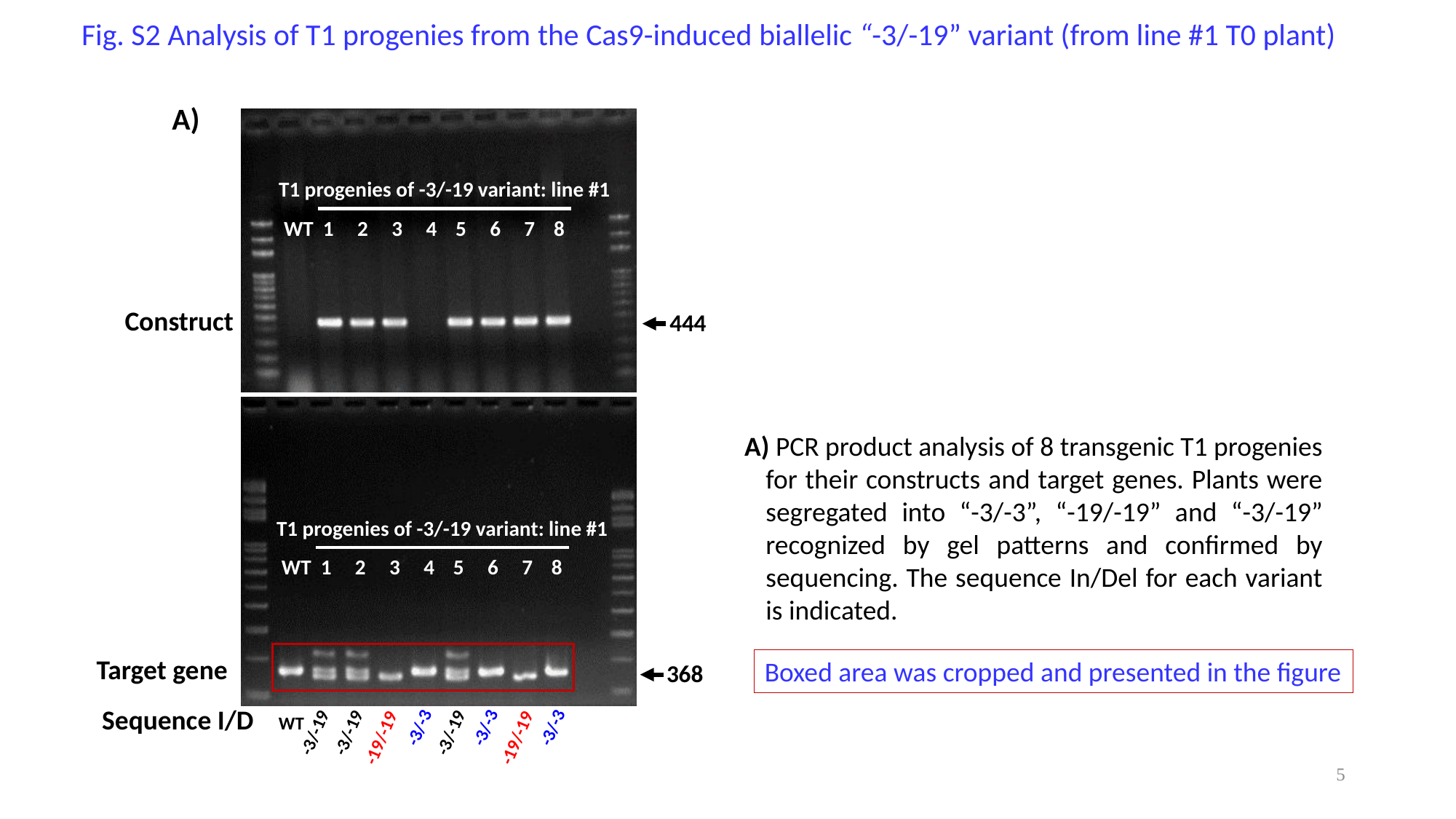

Fig. S2 Analysis of T1 progenies from the Cas9-induced biallelic “-3/-19” variant (from line #1 T0 plant)
A)
T1 progenies of -3/-19 variant: line #1
WT 1 2 3 4 5 6 7 8
Construct
444
T1 progenies of -3/-19 variant: line #1
WT 1 2 3 4 5 6 7 8
Target gene
368
Sequence I/D WT
-3/-3
-3/-3
-3/-3
-3/-19
-3/-19
-3/-19
-19/-19
-19/-19
A) PCR product analysis of 8 transgenic T1 progenies for their constructs and target genes. Plants were segregated into “-3/-3”, “-19/-19” and “-3/-19” recognized by gel patterns and confirmed by sequencing. The sequence In/Del for each variant is indicated.
Boxed area was cropped and presented in the figure
5

## Slide 6
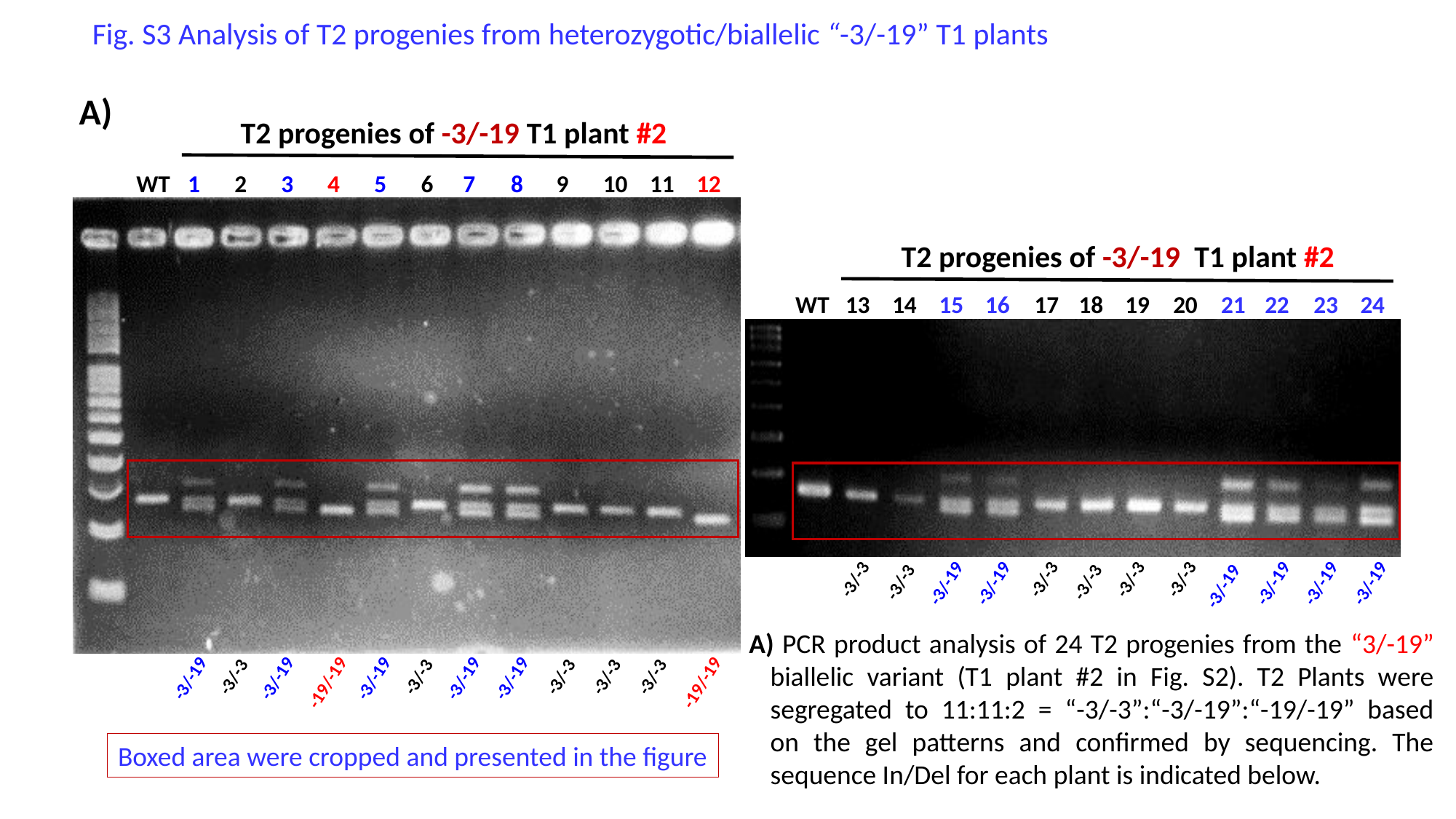

Fig. S3 Analysis of T2 progenies from heterozygotic/biallelic “-3/-19” T1 plants
A)
T2 progenies of -3/-19 T1 plant #2
WT
1
2
3
4
5
6
7
8
9
10
11
12
-3/-3
-3/-3
-3/-3
-3/-3
-3/-3
-3/-19
-3/-19
-3/-19
-3/-19
-3/-19
-19/-19
-19/-19
T2 progenies of -3/-19 T1 plant #2
WT
13
14
15
16
17
18
19
20
21
22
23
24
-3/-3
-3/-3
-3/-3
-3/-3
-3/-3
-3/-3
-3/-19
-3/-19
-3/-19
-3/-19
-3/-19
-3/-19
A) PCR product analysis of 24 T2 progenies from the “3/-19” biallelic variant (T1 plant #2 in Fig. S2). T2 Plants were segregated to 11:11:2 = “-3/-3”:“-3/-19”:“-19/-19” based on the gel patterns and confirmed by sequencing. The sequence In/Del for each plant is indicated below.
Boxed area were cropped and presented in the figure
